# MicroRNA-199a-3p inhibits ovarian cancer cell viability by targeting the oncogene YAP1

**DOI:** 10.3892/mmr.2022.12892

**Published:** 2022-11-07

**Authors:** Yanfang He, Xiangyang Yu, Yajuan Tang, Yanjuan Guo, Jinling Yuan, Jinghe Bai, Tao Yao, Xiongzhi Wu

Mol Med Rep 23: 237, 2021; DOI: 10.3892/mmr.2021.11876

Following the publication of the above article, the authors have submitted a request that it be retracted on account of the fact that, when requested to do so, the first author was unable to provide the original data for this article.

The Editor of *Molecular Medicine Reports* has agreed with the request that this article be retracted. Note that all the authors agree with the decision to retract this article. The Editor and the authors regret any inconvenience that this retraction will cause to the readership of the Journal.

